# The rationalization of carbon monoxide and hemoglobin association

**DOI:** 10.1371/journal.pone.0346152

**Published:** 2026-03-30

**Authors:** Chihjen Lee, Nikki Chen

**Affiliations:** 1 Cedars-Sinai Medical Center, Los Angeles, California, United States of America; 2 University of California, Los Angeles, California, United States of America; Versiti Blood Research Institute, UNITED STATES OF AMERICA

## Abstract

**Background/objective:**

Carbon monoxide (CO) is a colorless, odorless toxic gas responsible for approximately 100,000 emergency department visits and more than 420 deaths annually in the United States. Although CO–hemoglobin interactions have been extensively studied, a direct relationship between CO saturation and partial pressure has not been well established. This study aimed to derive a simple equation describing this relationship using principles analogous to the oxygen–hemoglobin association model.

**Methods:**

CO saturation was defined as the fraction of hemoglobin binding sites occupied by CO. Using chemical kinetics, the concentrations of CO, O₂, and hemoglobin, together with their association constants, were incorporated into the saturation formulation. Algebraic substitution and simplification yielded a rational function with four unknown coefficients. At a fixed oxygen partial pressure of 100 mmHg, four equilibrium (PCO, CO saturation) data points were used to solve for the coefficients of a fourth-degree rational function.

**Results:**

The derived CO–hemoglobin association equation reproduced the four derivation data points exactly and closely approximated additional literature values. The resulting association curve was hyperbolic. Fractional analysis demonstrated that Hb, HbCO, Hb(CO)₂, Hb(CO)₃, and Hb(CO)₄ fractions peaked at CO saturations of 0%, 25%, 50%, 75%, and 100%, respectively, with the triply bound form predominating overall.

**Conclusions:**

The CO–hemoglobin association could be described by a fourth-degree rational equation, enabling estimation of either CO saturation or CO partial pressure when one is known and providing a framework extendable to varying oxygen tensions.

## Introduction

Carbon Monoxide(CO) poisoning is a critical public health concern that is a leading cause of accidental toxic exposure and preventable deaths in the United States. It is a “silent killer” that poses a threat to health as it disrupts the ability of the body to deliver oxygen, causing hypoxia. Since the brain and heart require high levels of oxygen, hypoxia can lead to potentially permanent neurological and cardiovascular dysfunction and injury, and may ultimately result in death if left untreated. It triggers approximately 100,000 emergency department visits and over 420 accidental deaths in the United States each year.

CO has a much higher affinity for hemoglobin than oxygen, so even low concentrations of CO can disrupt oxygen transportation. Additionally, when CO binds to the ferrous iron in hemoglobin, the hemoglobin’s oxygen affinity also increases, which limits oxygen unloading in tissues. To assess CO poisoning, it is important to understand the relationship between CO saturation, hemoglobin, and the underlying binding dynamics.

Although there has been extensive research conducted on the interaction between CO and hemoglobin, current literature does not provide an equation to represent the relationship between CO saturation and its partial pressure. A form of the Coburn–Forster–Kane (CFK) equation is expressed as a differential equation that relates the rate of change of CO saturation to the mean capillary CO pressure. Although it is useful for evaluating CO uptake and removal, it is not designed for the rapid assessments required in clinical practice.

In 2023, Chou and Lee reported a theory of oxygen–hemoglobin association and derived a fourth-degree rational function equation based on clinical datasets [1]. Using an analogous approach to Chou and Lee, we sought to develop an equation expressing CO saturation as a function of CO partial pressure that would facilitate understanding of CO–hemoglobin binding behavior. Additionally, this formulation would allow for the calculation of the fractional saturation of each carboxyhemoglobin species.

In clinical practice, both arterial CO saturation and CO partial pressure can be measured in the laboratory; alternatively, pulse CO-oximetry allows continuous, noninvasive monitoring of CO saturation. Given such measurements, arterial PCO can be determined rapidly from the derived equation. Assuming an equilibrium oxygen partial pressure of 100 mmHg, the CO saturation equation was established by considering all four oxyhemoglobin and carboxyhemoglobin species, their various combinations, and unliganded hemoglobin. Using values extracted from the literature, four equilibrium data pairs for PCO and CO saturation were assembled, allowing four unknown coefficients to be solved from four independent linear equations [1]. The resulting coefficients were then rounded to simplify the expression. A step-by-step derivation is presented in the following section.

## Methods

### Derivation of the Carbon monoxide–hemoglobin association equation

Each hemoglobin molecule contains four heme groups and, consequently, four ferrous ions. Each ferrous ion can bind to either O₂ or CO, allowing a hemoglobin molecule to bind up to four ligands simultaneously. As a result, hemoglobin may exist in multiple species:


$$Hb,HbO_{\text{2}},Hb{\left(O_{\text{2}}\right)}_{\text{2}},Hb{\left(O_{\text{2}}\right)}_{\text{3}},Hb{\left(O_{\text{2}}\right)}_{\text{4}}$$

$$HbCO,Hb(CO{)}_{\text{2}},Hb(CO{)}_{\text{3}},Hb(CO{)}_{\text{4}}$$

$$HbO_{\text{2}}(CO),\;HbO_{\text{2}}(CO{)}_{\text{2}},\;HbO_{\text{2}}(CO{)}_{\text{3}}$$

$$\;Hb{\left(O_{\text{2}}\right)}_{\text{2}}(CO),\;Hb{\left(O_{\text{2}}\right)}_{\text{2}}(CO{)}_{\text{2}}$$

$$Hb{\left(O_{\text{2}}\right)}_{\text{3}}(CO)$$

A general representation of the hemoglobin association is:


$$Hb+iO_{\text{2}}+jCO↔Hb{\left(O_{\text{2}}\right)}_{i}(CO{)}_{j}$$

where 0≤i≤4, 0≤j≤4, and 0≤i+j≤4

According to the laws of chemical kinetics, the concentration of each species can be written as:


$$\left[Hb{\left(O_{\text{2}}\right)}_{i}(CO{)}_{j}\right]=\lambda_{i,j}[Hb]{\left(PO_{\text{2}}\right)}^{i}(PCO{)}^{j}$$ (1)

where $\lambda_{i,j}$ is the association constant.

Carbon monoxide saturation was defined as the ratio of hemoglobin binding sites occupied by CO to the total number of available binding sites:


$$CO\;sat=\frac{j[Hb{\left(O_{\text{2}}\right)}_{i}(CO{)}_{j}]}{\text{4}[Hb{\left(O_{\text{2}}\right)}_{i}(CO{)}_{j}]}$$

Substituting $\left[Hb{\left(O_{\text{2}}\right)}_{i}(CO{)}_{j}\right]$ with $\lambda_{i,j}[Hb]{\left(PO_{\text{2}}\right)}^{i}(PCO{)}^{j}$ from equation (1) yielded:


$$CO\;sat=\frac{j\lambda_{i,j}[Hb]{\left(PO_{\text{2}}\right)}^{i}(PCO{)}^{j}}{\text{4}\lambda_{i,j}[Hb]{\left(PO_{\text{2}}\right)}^{i}(PCO{)}^{j}}$$ (2)

The hemoglobin term canceled, giving:


$$CO\;sat=\frac{j\lambda_{i,j}{\left(PO_{\text{2}}\right)}^{i}(PCO{)}^{j}}{\text{4}\lambda_{i,j}{\left(PO_{\text{2}}\right)}^{i}(PCO{)}^{j}}$$ (3)

Since i, j, $\lambda_{i,j}$ and $PO_{\text{2}}$ were constants, the following final *CO sat* equation could be derived. (See S1 Appendix for detailed steps.)


$$CO\;sat=\frac{a_{\text{1}}(PCO)+\text{2}a_{\text{2}}(PCO{)}^{\text{2}}+\text{3}a_{\text{3}}(PCO{)}^{\text{3}}+\text{4}a_{\text{4}}(PCO{)}^{\text{4}}}{\text{4}+\text{4}a_{\text{1}}(PCO)+\text{4}a_{\text{2}}(PCO{)}^{\text{2}}+\text{4}a_{\text{3}}(PCO{)}^{\text{3}}+\text{4}a_{\text{4}}(PCO{)}^{\text{4}}}$$ (4)

Where


$$a_{j}=\frac{\lambda_{i,j}{\left(PO_{\text{2}}\right)}^{i}}{\lambda_{i,\text{0}}{\left(PO_{\text{2}}\right)}^{i}}$$

(See S1 Appendix for detailed explanations.)

At an oxygen partial pressure of 100 mmHg, $\lambda_{i,j}$ was constant, and $a_{j}$ represented a set of coefficients at this condition. At other PO₂ values, the coefficients would differ, resulting in rightward or leftward shifts of the CO–hemoglobin association curve.

Equation (4) was solvable when four (PCO, CO saturation) data points were provided, previously demonstrated by Chou and Lee [1]. Four data points obtained from the literature were therefore selected and used as the derivation dataset; these points were reproduced exactly by construction. To maximize representativeness, the selected four data points were evenly distributed across the expected range of carbon monoxide saturation, thereby capturing the overall shape of the association curve. The remaining four literature-derived data points, which were not used in model development, were reserved a priori for validation. Model performance was assessed by comparing predicted values with published values using absolute and percent error.

## Results

Equilibrium data points for carbon monoxide partial pressure (PCO) and corresponding saturations were identified from the literature as reported by Hess et al. [2]. PCO values were expressed in parts per thousand (ppt). To ensure representativeness, four data points were selected—highlighted in bold—such that they were evenly distributed across the expected range of CO saturation, thereby capturing the overall shape of the association curve:

Table 1 CO saturations corresponding to CO partial pressure from previously published data.

**Table 1:** CO partial pressure and saturation.

CO partial pressure (ppt)	CO saturation
0.0087	1.71%
**0.025**	**4.21%**
0.035	5.82%
0.050	7.79%
**0.1**	**14.83%**
0.2	25.96%
**0.5**	**46.61%**
**1**	**63.58%**

These four data points were substituted into equation (4), yielding the following coefficients:


$$a_{\text{1}}\approx \text{7}.\text{0}\text{7}\text{7}\text{2},\;a_{\text{2}}\approx \text{1}\text{7}.\text{7}\text{1}\text{2}\text{4},\;a_{\text{3}}\approx \text{2}\text{2}.\text{0}\text{5}\text{3}\text{7},\;a_{\text{4}}\approx \text{8}.\text{9}\text{3}\text{1}\text{9}$$

Thus, the CO–hemoglobin association equation was expressed as:


$$CO\;sat(\%)=\frac{\text{1}}{\text{4}}\times \frac{\text{7}.\text{0}\text{7}\text{7}\text{2}(PCO)+\text{3}\text{5}.\text{4}\text{2}\text{4}\text{8}(PCO{)}^{\text{2}}+\text{6}\text{6}.\text{1}\text{6}\text{1}\text{1}(PCO{)}^{\text{3}}+\text{3}\text{5}.\text{7}\text{2}\text{7}\text{6}(PCO{)}^{\text{4}}}{\text{1}+\text{7}.\text{0}\text{7}\text{7}\text{2}(PCO)+\text{1}\text{7}.\text{7}\text{1}\text{2}\text{4}(PCO{)}^{\text{2}}+\text{2}\text{2}.\text{0}\text{5}\text{3}\text{7}(PCO{)}^{\text{3}}+\text{8}.\text{9}\text{3}\text{1}\text{9}(PCO{)}^{\text{4}}}$$

Because the equilibrium data points were approximate, the coefficients were rounded to improve practicality for application. The resulting simplified equation was:


$$CO\;sat(\%)=\frac{\text{1}}{\text{4}}\times \frac{\text{7}(PCO)+\text{3}\text{6}(PCO{)}^{\text{2}}+\text{6}\text{6}(PCO{)}^{\text{3}}+\text{3}\text{6}(PCO{)}^{\text{4}}}{\text{1}+\text{7}(PCO)+\text{1}\text{8}(PCO{)}^{\text{2}}+\text{2}\text{2}(PCO{)}^{\text{3}}+{\text{9}(PCO)}^{\text{4}}}$$

with PCO expressed in ppt.

These two equations produced nearly indistinguishable curves when plotted.

From the original equation, a tabulated set of values was generated, demonstrating a close fit with the data reported by Hess et al. [2].

Table 2 CO saturation values at varying CO partial pressures, comparing values predicted by the derived equation with previously published data. The derived equation reproduced the four derivation data points exactly, as expected. When applied to the four independent validation points, predicted CO saturation closely approximated published values across the examined range with a mean absolute error approximately 0.17% and a mean percent error approximately 4.0%. The largest relative error occurred at very low saturation, where small absolute differences inflated percent error.

**Table 2:** Carbon monoxide saturation at different partial pressures.

CO in %	CO in ppm	CO in ppt	CO sat from equation	CO sat in data points	Absolute error (%)	Percent error (%)
0.00087%	8.7	0.0087	1.51%	1.71%	−0.20	−11.7%
0.0025%	25	0.025	**4.21%**	**4.21%**		
0.0035%	35	0.035	5.79%	5.82%	−0.03	−0.52%
0.005%	50	0.05	8.05%	7.79%	+0.26	+3.34%
0.01%	100	0.1	**14.83**%	**14.83**%		
0.02%	200	0.2	25.79%	25.96%	−0.17	−0.65%
0.05%	500	0.5	**46.61%**	**46.61%**		
0.1%	1000	1	**63.58%**	**63.58%**		

**Fig 1:**
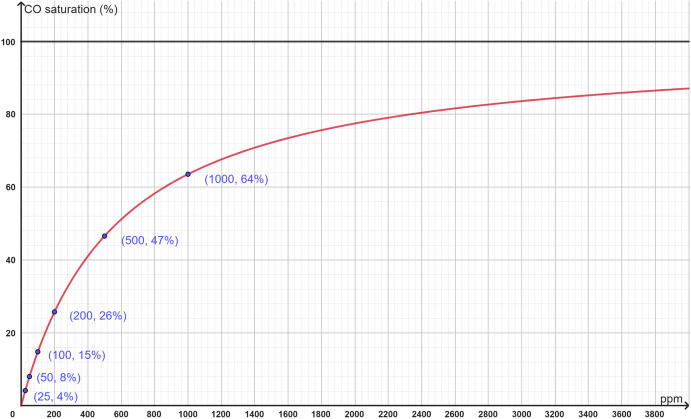
Plot of the CO–hemoglobin saturation equation. The x-axis represents CO partial pressure (ppm), and the y-axis represents CO saturation (%). The curve demonstrates the characteristic hyperbolic decline in slope.

The plot of the derived equation (Fig 1) exhibited a hyperbolic shape, consistent with previous findings [3]. This curve was characterized by a continuous decline in slope, a defining feature of hyperbolic association relationships.

### Breakdown of each carboxyhemoglobin species

Unlike other models described in the literature, the present equation enabled determination of the fractional distribution of the five carboxyhemoglobin species. In each case, the denominator represented the sum of all carboxyhemoglobin species, whereas the numerator corresponded to the specific species of interest:


$$Hb(CO{)}_{k}\text{ fraction}=\frac{\left[Hb{\left(O_{\text{2}}\right)}_{i}(CO{)}_{k}\right]}{\left[Hb{\left(O_{\text{2}}\right)}_{i}(CO{)}_{j}\right]},\;k=\text{0},\;\text{1},\;\text{2},\;\text{3},or\;\text{4}$$

By comparing the above equation to the CO saturation equation,


$$CO\;sat=\frac{j[Hb{\left(O_{\text{2}}\right)}_{i}(CO{)}_{j}]}{\text{4}[Hb{\left(O_{\text{2}}\right)}_{i}(CO{)}_{j}]}$$

the following fractional equations were derived:


$$Hb\;fraction=\frac{\text{1}}{\text{1}+\text{7}(PCO)+\text{1}\text{8}(PCO{)}^{\text{2}}+\text{2}\text{2}(PCO{)}^{\text{3}}+\text{9}(PCO{)}^{\text{4}}}$$

$$HbCO\;fraction=\frac{\text{7}(PCO)}{\text{1}+\text{7}(PCO)+\text{1}\text{8}(PCO{)}^{\text{2}}+\text{2}\text{2}(PCO{)}^{\text{3}}+\text{9}(PCO{)}^{\text{4}}}$$

$$Hb(CO{)}_{\text{2}}\;fraction=\frac{\text{1}\text{8}(PCO{)}^{\text{2}}}{\text{1}+\text{7}(PCO)+\text{1}\text{8}(PCO{)}^{\text{2}}+\text{2}\text{2}(PCO{)}^{\text{3}}+\text{9}(PCO{)}^{\text{4}}}$$

$$Hb(CO{)}_{\text{3}}\;fraction=\frac{\text{2}\text{2}(PCO{)}^{\text{3}}}{\text{1}+\text{7}(PCO)+\text{1}\text{8}(PCO{)}^{\text{2}}+\text{2}\text{2}(PCO{)}^{\text{3}}+\text{9}(PCO{)}^{\text{4}}}$$

$$Hb(CO{)}_{\text{4}}\;fraction=\frac{\text{9}(PCO{)}^{\text{4}}}{\text{1}+\text{7}(PCO)+\text{1}\text{8}(PCO{)}^{\text{2}}+\text{2}\text{2}(PCO{)}^{\text{3}}+\text{9}(PCO{)}^{\text{4}}}$$

**Fig 2:**
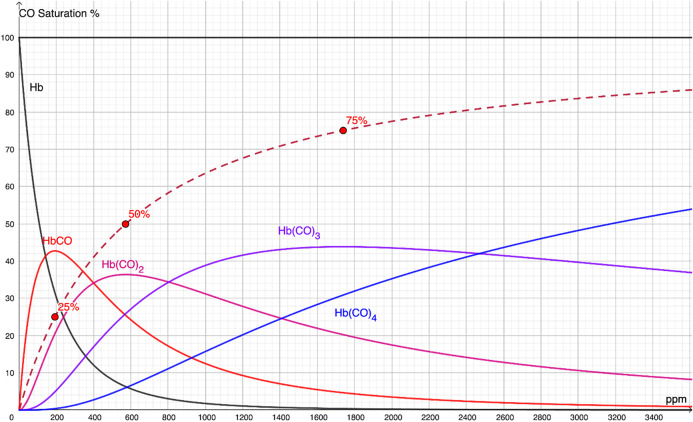
Fractional distributions of carboxyhemoglobin species (Hb, HbCO, Hb(CO)₂, Hb(CO)₃, and Hb(CO)₄), with the CO saturation curve superimposed. Triply bound carboxyhemoglobin was the dominant species at pressures between 800 ppm and 2440 ppm. Each species fraction peaked at CO saturations of 0%, 25%, 50%, 75%, and 100%, respectively.

The plots of these fractions, together with the overall CO saturation curve, are presented in Fig 2.

At minimal CO pressure, only a small fraction of HbCO was present. With increasing PCO, hemoglobin bound progressively more carbon monoxide, forming singly and doubly bound species. Above 800 ppm, triply bound carboxyhemoglobin became the dominant form. The fraction of quadruple-bound carboxyhemoglobin increased nearly linearly, without evidence of the cooperative binding that characterizes oxygen binding.

The distribution patterns differed markedly from those of oxyhemoglobin. For oxygen, the triply bound species exhibited the lowest fraction, whereas for carbon monoxide, the triply bound species was most abundant. Perrella et al. investigated intermediates of the CO–hemoglobin interaction using deoxyhemoglobin [4]. Although their reported distribution curves differed from those of the present study, they clearly demonstrated that singly, doubly, and triply bound carboxyhemoglobin fractions peak at CO saturations of 25%, 50%, and 75%, respectively. This behavior is a unique property of fourth-degree rational association equations and can be proven mathematically. The laboratory findings of Perrella et al. therefore provide indirect validation of the present model.

## Discussion

Chou and Lee recently derived the oxygen–hemoglobin association equation [1]. The present study applied a similar, though more complex, approach to establish the carbon monoxide–hemoglobin association equation.

Carbon monoxide is not detectable by human senses; however, at elevated concentrations it can rapidly cause respiratory distress. Buchelli et al. reported that approximately 20% of an unselected population exhibited elevated COHb levels, defined as ≥2.5% in nonsmokers and ≥5% in smokers [5]. Among these individuals, 78% were nonsmokers (mean COHb 3.2%), while 22% were smokers (mean COHb 6.7%) [5]. Their study concluded that domestic environments were the most likely source of exposure [5].

Environmental and clinical sources of carbon monoxide include volcanic activity, bushfires, and automobile emissions. In anesthetic practice, it is well established that desflurane in contact with desiccated CO₂ absorbents (e.g., Baralyme) can generate significant concentrations of carbon monoxide. Stabernack et al. documented concentrations as high as 36,000 ppm under these conditions [6]. Berry et al. subsequently reported a case of severe intraoperative carbon monoxide poisoning in which the patient reached a COHb level of 36% during anesthesia with desflurane and Baralyme [7]. Although arterial CO pressure was not reported in that case, application of the present equation estimated the corresponding PCO to be 323.16 ppm.

Table 3 summarizes carbon monoxide concentrations under selected environmental and clinical conditions, along with the corresponding maximum saturations derived using the present model.

**Table 3:** Carbon monoxide saturation in different environments.

Source	CO concentration in environment	CO sat from our equation
Earth’s atmosphere [8]	100 ppb	0.02%
Federal standard	9 ppm	1.56%
Typical urban area	10 ppm	1.73%
Heavy traffic area	50 ppm	8.05%
Volcanic gases, lower limit	0.01%	14.83%
Volcanic gases, upper limit	2%	97.04%
Bushfires observers [9]	11 ppm	1.90%
Nonsmoking firefighters [9]	17 ppm	2.91%
Smoking firefighters [9]	31 ppm	5.16%
Desflurane and Baralyme [7]	323.16 ppm	36%

Table 3. Carbon monoxide saturations calculated from the derived equation based on partial pressures in different environments. As shown in the first row, the atmospheric partial pressure of CO is approximately 100 ppb, corresponding to a predicted CO saturation of 0.02%.

### Coburn–Forster–Kane (CFK) equation

The Coburn–Forster–Kane (CFK) equation, first introduced in 1965, has been widely applied to estimate carbon monoxide absorption, excretion, and hemoglobin saturation under varying conditions of environmental CO concentration, exposure duration, and alveolar ventilation [10–12]. However, the CFK equation does not directly express CO saturation as a function of CO partial pressure. The present model complements the CFK framework by providing additional insight into CO–hemoglobin binding behavior, analogous to the oxygen–hemoglobin association.

The CFK equation can be expressed as the following differential equation [10,11]:


$$\frac{d[CO\;sat]}{dt}=k(PiCO-mean\_capPCO)$$

Where *PiCO* denotes inspired CO pressure and *mean_capPCO* represents the mean capillary CO pressure. The proportionality constant *k* depends on several physiological factors, including endogenous CO production, blood volume, CO diffusion capacity, barometric pressure corrected for water vapor, and alveolar ventilation [11].

This formulation indicates that the rate of change in CO saturation approaches zero as mean-capillary PCO approaches the inspired PCO, corresponding to the plateau observed in CO saturation curves. The data points used in the present study were estimated from the CO saturation versus CO exposure plot reported by Hess et al. [2], where equilibrium saturation values had been reached.

While the CFK equation describes CO saturation as a function of exposure conditions, the present model expresses saturation directly as a function of PCO at a fixed PO₂ of 100 mmHg, with the potential to extend to other oxygen pressures (see CO Association Curves at Different Oxygen Pressures).

### Relative affinity of carbon monoxide and oxygen for hemoglobin

Historically, the following reaction has been used to investigate the relative affinity of hemoglobin for carbon monoxide compared with oxygen:


$$HbO_{\text{2}}+CO↔HbCO+O_{\text{2}}$$

with the equilibrium constant expressed as:


$$K=\frac{\left[HbCO\right]}{\left[HbO_{\text{2}}\right]}\times \frac{PO_{\text{2}}}{PCO}$$

When the concentrations of HbCO and HbO₂ are equal, those two terms cancel, yielding:


$$K=\frac{PO_{\text{2}}}{PCO}$$

Previous studies reported values of *K* ranging from 200 to 490 [12–14]. However, this formulation oversimplifies hemoglobin binding, as hemoglobin possesses four binding sites and thus forms four distinct oxyhemoglobin and four distinct carboxyhemoglobin species. The conventional notations [HbCO] and [HbO₂] therefore do not fully represent the complexity of CO and O₂ binding.

The present equation allows a more precise evaluation of relative affinity by calculating the ratio $\frac{PO_{\text{2}}}{PCO}$ at the point where both CO and O₂ saturations equal 50%.

Setting CO saturation to 50%:


$$Let\;CO\;sat=\frac{\text{1}}{\text{4}}\times \frac{\text{7}.\text{0}\text{7}\text{7}\text{2}(PCO)+\text{3}\text{5}.\text{4}\text{2}\text{4}\text{8}(PCO{)}^{\text{2}}+\text{6}\text{6}.\text{1}\text{6}\text{1}\text{1}(PCO{)}^{\text{3}}+\text{3}\text{5}.\text{7}\text{2}\text{7}\text{6}(PCO{)}^{\text{4}}}{\text{1}+\text{7}.\text{0}\text{7}\text{7}\text{2}(PCO)+\text{1}\text{7}.\text{7}\text{1}\text{2}\text{4}(PCO{)}^{\text{2}}+\text{2}\text{2}.\text{0}\text{5}\text{3}\text{7}(PCO{)}^{\text{3}}+\text{8}.\text{9}\text{3}\text{1}\text{9}(PCO{)}^{\text{4}}}=\text{0}.\text{5}$$

After solving the above equation, PCO ≈0.5724 ppt

Because CO saturation was fixed at 50%, O₂ saturation can reasonably be assumed to also equal 50% under equilibrium conditions. Given that the model was derived at an oxygen pressure of 100 mmHg, the relative affinity is:


$$\frac{PO_{\text{2}}}{PCO}=\frac{\text{1}\text{0}\text{0}}{\frac{\text{0}.\text{5}\text{7}\text{2}\text{4}}{\text{1}\text{0}\text{0}\text{0}}\times \text{7}\text{6}\text{0}}≅\text{2}\text{3}\text{0}$$

Thus, the present model confirms that the affinity of carbon monoxide for hemoglobin is approximately 230 times greater than that of oxygen.

### Hyperbola versus sigmoidal association curves

The carbon monoxide–hemoglobin association curve has been demonstrated to be hyperbolic [3], a finding confirmed by the present equation. The hyperbolic shape reflects a continuous decline in slope, indicating that progressively greater increases in carbon monoxide concentration are required to achieve the same incremental increase in hemoglobin saturation.

In contrast, the oxygen–hemoglobin association curve exhibits a sigmoidal configuration. The initial upward concavity at low oxygen pressures reflects cooperative binding, whereby the binding of the first oxygen molecules facilitates subsequent binding events. At higher oxygen pressures, the curve transitions to downward concavity as saturation approaches its maximum.

These distinct curve shapes highlight fundamental physiological differences: oxygen binding is optimized for efficient uptake at low partial pressures, while carbon monoxide binding is characterized by high initial affinity but limited cooperativity, thereby resisting excessive binding at higher concentrations.

### CO association curves at different oxygen pressures

The present equation was derived at a fixed oxygen partial pressure (PO₂) of 100 mmHg. At other oxygen pressures, the coefficients of the equation will vary, resulting in distinct association curves. As shown below, the coefficient $a_{j}$ is a function of PO₂.


$$a_{j}=\frac{\lambda_{i,j}{\left(PO_{\text{2}}\right)}^{i}}{\lambda_{i,\text{0}}{\left(PO_{\text{2}}\right)}^{i}}$$

In theory, substituting different values of PO₂ yields new sets of $a_{j}$ coefficients and, consequently, different association curves. To compute these, the underlying association constants (λ) must first be determined, as they remain constant across different oxygen pressures.

### Strategy for solving the general association equation

1) At each PO₂ level, obtain four clinical data points of PCO and the corresponding carboxyhemoglobin saturations.
2) Use these data points to solve for the coefficients $a_{j}$.
3) Once the $a_{j}$ values are determined, solve for the association constants (λ). For example:

$$a_{\text{4}}=\frac{\lambda_{i,\text{4}}{\left(PO_{\text{2}}\right)}^{i}}{\lambda_{i,\text{0}}{\left(PO_{\text{2}}\right)}^{i}}=\frac{\lambda_{\text{0},\text{4}}}{\text{1}+\lambda_{\text{1},\text{0}}{\left(PO_{\text{2}}\right)}^{\text{1}}+\lambda_{\text{2},\text{0}}{\left(PO_{\text{2}}\right)}^{\text{2}}+\lambda_{\text{3},\text{0}}{\left(PO_{\text{2}}\right)}^{\text{3}}+\lambda_{\text{4},\text{0}}{\left(PO_{\text{2}}\right)}^{\text{4}}}$$

4) As shown above, there are five λ values to be solved. Therefore, five equations are needed, each using a different pair of values of $a_{\text{4}}$ and $PO_{\text{2}}$
5) Repeat similar procedures for $a_{\text{1}},\;a_{\text{2}}\text{ and }a_{\text{3}}$ to derive all association constants (λ).

Once all λ values are determined, the general carbon monoxide hemoglobin association equation can be established. This general equation incorporates both PO₂ and PCO as variables, enabling calculation of CO saturation across a range of oxygen pressure conditions.

A detailed mathematical exposition is provided in S2 Appendix.

### Spectrophotometry

In clinical practice, spectrophotometry is widely used to estimate the carbon monoxide saturation of hemoglobin. Both direct spectrophotometry and CO pulse oximetry rely on the differential absorption of light at specific wavelengths by hemoglobin species, thereby enabling calculation of the ratio of carboxyhemoglobin to total hemoglobin [15]. CO pulse oximetry depends on pulsatile peripheral perfusion and may be unreliable in patients with impaired peripheral circulation, hypothermia, or burn injuries. Its accuracy can also be influenced by external factors such as ambient light. Direct spectrophotometry remains the gold standard for measuring carbon monoxide saturation, although laboratory errors may occasionally occur due to sample mislabeling, mishandling, or delays in specimen transport.

The equation derived in this study provides a theoretical framework for estimating carboxyhemoglobin saturation based on environmental carbon monoxide exposure. While it is not intended to replace spectrophotometry, this model offers a complementary conceptual tool that describes the relationship between carbon monoxide partial pressure and hemoglobin saturation. Such a framework may facilitate a deeper understanding of carbon monoxide binding dynamics, analogous to the well-characterized oxygen–hemoglobin interaction.

### Clinical applications

As an example of clinical application, Table 2 presents carbon monoxide partial pressures observed under different environmental and clinical conditions. Assuming maximal exposure, the corresponding potential maximum CO saturations were calculated using the present equation, which provides a direct relationship between arterial CO partial pressure and hemoglobin saturation.

While spectrophotometry and pulse CO-oximetry measure CO saturation, the proposed equation enables inverse estimation of arterial CO partial pressure under normal physiological conditions. Discrepancies between measured and predicted values may indicate altered respiratory physiology, such as the presence of intrapulmonary or intracardiac shunt.

Beyond carbon monoxide, this methodological framework may be extended to other complex ligand–receptor binding systems in biology and medicine. Analogous equations could be constructed to solve for association constants using only clinical saturation data, thereby offering a generalizable approach to studying binding dynamics.

### Limitations

Although this approach is grounded in chemical kinetics and mathematical modeling, it relies heavily on the accuracy of input data. Because the method requires four equilibrium data points to solve for the model coefficients, the choice of points directly influences the resulting equation. Selecting an alternative set of four points will yield slightly different coefficients. Incorporating more than four points into the system leads to an overdetermined set of equations, which is generally unsolvable—an inherent limitation of linear algebra. Thus, the model can exactly fit only four data points.

To maximize representativeness, four data points were selected that were evenly distributed across the expected range of carbon monoxide saturation, thereby capturing the overall shape of the association curve. While the framework adheres strictly to chemical kinetics and algebraic principles, clinical measurements may diverge from theoretical predictions due to experimental error, interindividual variability, or other physiological factors. Nevertheless, the model approximates the fundamental characteristics of carbon monoxide binding to hemoglobin.

As shown in Table 1, the four selected data points are matched exactly, while additional values exhibit close agreement. The dataset used in this study was derived from published literature [3,16]. Further validation could be achieved by directly measuring arterial carbon monoxide partial pressures and corresponding hemoglobin saturations in controlled experimental settings. Such datasets would likely produce equations with different coefficients, reflecting natural variability in empirical measurements.

It is also important to recognize that hemoglobin variants may differ in their affinity for carbon monoxide. In adults, the predominant form is hemoglobin A (HbA, α₂β₂), which constitutes approximately 95–98% of total hemoglobin. Hemoglobin A₂ (HbA₂, α₂δ₂) accounts for 2–3%, while fetal hemoglobin (HbF, α₂γ₂) persists at low levels after birth. Hemoglobin S (HbS, α₂β^s^₂) is present in individuals with sickle cell disease and contains abnormal β-globin chains. The equations derived in this study apply specifically to normal hemoglobin composition. However, if appropriate datasets are available, the same methodology could be extended to derive alternative equations for individuals with sickle cell disease, newborns with elevated HbF, or other hemoglobin variants.

### Generative AI

During the preparation of this manuscript, ChatGPT was used to improve English language clarity and grammar. After using the tool, all content was carefully reviewed and revised by the authors, who take full responsibility for the final version of the manuscript.

## Supporting information

S1 File Source of data. (DOCX)

S1 Appendix Deriving Equation (4) from Equation (3). (DOCX)

S2 Appendix Deriving the General Equation. (DOCX)
