# Supplementary material for: The rationalization of carbon monoxide and hemoglobin association
Source: PLoS One. 2026 Mar 30;21(3):e0346152. doi: 10.1371/journal.pone.0346152 (PMC13035115; doi:10.1371/journal.pone.0346152)
Supplement: S1 File — (DOCX) [file pone.0346152.s001.docx]

**Supporting Information**

The data points for carbon monoxide partial pressure (PCO) and corresponding saturations were extracted from:

Hess DR. Inhaled Carbon Monoxide: From Toxin to Therapy. Respir Care. 2017 Oct;62(10):1333-1342. doi: 10.4187/respcare.05781. Epub 2017 Aug 14. PMID: 28807985.
