# Supplementary material for: The rationalization of carbon monoxide and hemoglobin association
Source: PLoS One. 2026 Mar 30;21(3):e0346152. doi: 10.1371/journal.pone.0346152 (PMC13035115; doi:10.1371/journal.pone.0346152)
Supplement: S1 Appendix — (DOCX) [file pone.0346152.s002.docx]

**Appendix I: Deriving equation (4) from equation (3)**

Listed below is equation (3).

|  | $CO sat=\frac{j\lambda_{i,j}\left( PO_{2} \right)^{i}\left( PCO \right)^{j}}{4\lambda_{i,j}\left( PO_{2} \right)^{i}\left( PCO \right)^{j}}$ | ----- Eq. 3 |
| --- | --- | --- |

Expressed with the $\Sigma$ symbol, equation (3) became the following equation.

$$CO sat=\frac{j\left[ \sum_{i=0}^{4-j} \lambda_{i,j}\left( PO_{2} \right)^{i} \right]\left( PCO \right)^{j}}{4\left[ \sum_{i=0}^{4-j} \lambda_{i,j}\left( PO_{2} \right)^{i} \right]\left( PCO \right)^{j}}$$

Let $b_{j}=\sum_{i=0}^{4-j} \lambda_{i,j}\left( PO_{2} \right)^{i}$

Then,

$$CO sat=\frac{j b_{j}\left( PCO \right)^{j}}{4 b_{j}\left( PCO \right)^{j}}$$

That was,

$$CO sat=\frac{b_{1}\left( PCO \right)^{1}+{2b}_{2}\left( PCO \right)^{2}+{3b}_{3}\left( PCO \right)^{3}+4b_{4}\left( PCO \right)^{4}}{4b_{0}+ {4b}_{1}\left( PCO \right)^{1}+{4b}_{2}\left( PCO \right)^{2}+{4b}_{3}\left( PCO \right)^{3}+4b_{4}\left( PCO \right)^{4}}$$

This equation could be further simplified as

|  | $CO sat=\frac{a_{1}\left( PCO \right)+2a_{2}\left( PCO \right)^{2}+3a_{3}\left( PCO \right)^{3}+4a_{4}\left( PCO \right)^{4}}{4+4a_{1}\left( PCO \right)+4a_{2}\left( PCO \right)^{2}+4a_{3}\left( PCO \right)^{3}+4a_{4}\left( PCO \right)^{4}}$ | ----- Eq. 4 |
| --- | --- | --- |

Where $a_{j}=\frac{b_{j}}{b_{0}}=\frac{\sum_{i=0}^{4-j} \lambda_{i,j}\left( PO_{2} \right)^{i}}{\sum_{i=0}^{4} \lambda_{i,0}\left( PO_{2} \right)^{i}}$

After simplifying the notation,

$$a_{j}=\frac{\lambda_{i,j}\left( PO_{2} \right)^{i}}{\lambda_{i,0}\left( PO_{2} \right)^{i}}$$

Listed below are $\lambda_{0,0}$ and expanded $a_{j}$ :

1. $\lambda_{0,0}=1$
2. $a_{0}=1$
3. $a_{1}=\frac{\lambda_{i,1}\left( PO_{2} \right)^{i}}{\lambda_{i,0}\left( PO_{2} \right)^{i}}=\frac{\lambda_{0,1}+\lambda_{1,1}\left( PO_{2} \right)^{1}+\lambda_{2,1}\left( PO_{2} \right)^{2}+\lambda_{3,1}\left( PO_{2} \right)^{3}}{1+\lambda_{1,0}\left( PO_{2} \right)^{1}+\lambda_{2,0}\left( PO_{2} \right)^{2}+\lambda_{3,0}\left( PO_{2} \right)^{3}+\lambda_{4,0}\left( PO_{2} \right)^{4}}$
4. $a_{2}=\frac{\lambda_{i,2}\left( PO_{2} \right)^{i}}{\lambda_{i,0}\left( PO_{2} \right)^{i}}=\frac{\lambda_{0,2}+\lambda_{1,2}\left( PO_{2} \right)^{1}+\lambda_{2,2}\left( PO_{2} \right)^{2}}{1+\lambda_{1,0}\left( PO_{2} \right)^{1}+\lambda_{2,0}\left( PO_{2} \right)^{2}+\lambda_{3,0}\left( PO_{2} \right)^{3}+\lambda_{4,0}\left( PO_{2} \right)^{4}}$
5. $a_{3}=\frac{\lambda_{i,3}\left( PO_{2} \right)^{i}}{\lambda_{i,0}\left( PO_{2} \right)^{i}}=\frac{\lambda_{0,3}+\lambda_{1,3}\left( PO_{2} \right)^{1}}{1+\lambda_{1,0}\left( PO_{2} \right)^{1}+\lambda_{2,0}\left( PO_{2} \right)^{2}+\lambda_{3,0}\left( PO_{2} \right)^{3}+\lambda_{4,0}\left( PO_{2} \right)^{4}}$
6. $a_{4}=\frac{\lambda_{i,4}\left( PO_{2} \right)^{i}}{\lambda_{i,0}\left( PO_{2} \right)^{i}}=\frac{\lambda_{0,4}}{1+\lambda_{1,0}\left( PO_{2} \right)^{1}+\lambda_{2,0}\left( PO_{2} \right)^{2}+\lambda_{3,0}\left( PO_{2} \right)^{3}+\lambda_{4,0}\left( PO_{2} \right)^{4}}$

As shown above, *aⱼ* was a function of PO₂.
