# Supplementary material for: The rationalization of carbon monoxide and hemoglobin association
Source: PLoS One. 2026 Mar 30;21(3):e0346152. doi: 10.1371/journal.pone.0346152 (PMC13035115; doi:10.1371/journal.pone.0346152)
Supplement: S2 Appendix — (DOCX) [file pone.0346152.s003.docx]

**Appendix II: Deriving the general equation**

From equation (4),

$$CO sat=\frac{a_{1}\left( PCO \right)+2a_{2}\left( PCO \right)^{2}+3a_{3}\left( PCO \right)^{3}+4a_{4}\left( PCO \right)^{4}}{4+4a_{1}\left( PCO \right)+4a_{2}\left( PCO \right)^{2}+4a_{3}\left( PCO \right)^{3}+4a_{4}\left( PCO \right)^{4}}$$

Theoretically, the coefficients $a_{j}$ are functions of the oxygen partial pressure ($PO_{2}$), as shown below. In these equations, $i \mathrm{ranges} \mathrm{from} 0 \mathrm{to} 4$, but is constrained by $i+j\leq4$, $j$ being the second index.

1. $a_{1}=\frac{\lambda_{i,1}\left( PO_{2} \right)^{i}}{\lambda_{i,0}\left( PO_{2} \right)^{i}}=\frac{\lambda_{0,1}+\lambda_{1,1}\left( PO_{2} \right)^{1}+\lambda_{2,1}\left( PO_{2} \right)^{2}+\lambda_{3,1}\left( PO_{2} \right)^{3}}{1+\lambda_{1,0}\left( PO_{2} \right)^{1}+\lambda_{2,0}\left( PO_{2} \right)^{2}+\lambda_{3,0}\left( PO_{2} \right)^{3}+\lambda_{4,0}\left( PO_{2} \right)^{4}}$
2. $a_{2}=\frac{\lambda_{i,2}\left( PO_{2} \right)^{i}}{\lambda_{i,0}\left( PO_{2} \right)^{i}}=\frac{\lambda_{0,2}+\lambda_{1,2}\left( PO_{2} \right)^{1}+\lambda_{2,2}\left( PO_{2} \right)^{2}}{1+\lambda_{1,0}\left( PO_{2} \right)^{1}+\lambda_{2,0}\left( PO_{2} \right)^{2}+\lambda_{3,0}\left( PO_{2} \right)^{3}+\lambda_{4,0}\left( PO_{2} \right)^{4}}$
3. $a_{3}=\frac{\lambda_{i,3}\left( PO_{2} \right)^{i}}{\lambda_{i,0}\left( PO_{2} \right)^{i}}=\frac{\lambda_{0,3}+\lambda_{1,3}\left( PO_{2} \right)^{1}}{1+\lambda_{1,0}\left( PO_{2} \right)^{1}+\lambda_{2,0}\left( PO_{2} \right)^{2}+\lambda_{3,0}\left( PO_{2} \right)^{3}+\lambda_{4,0}\left( PO_{2} \right)^{4}}$
4. $a_{4}=\frac{\lambda_{i,4}\left( PO_{2} \right)^{i}}{\lambda_{i,0}\left( PO_{2} \right)^{i}}=\frac{\lambda_{0,4}}{1+\lambda_{1,0}\left( PO_{2} \right)^{1}+\lambda_{2,0}\left( PO_{2} \right)^{2}+\lambda_{3,0}\left( PO_{2} \right)^{3}+\lambda_{4,0}\left( PO_{2} \right)^{4}}$

In this study, all data are obtained at $PO_{2}=100\mathrm{mmHg}$. The same procedure can be repeated at additional oxygen partial pressures – e.g., $PO_{2} values of 200, 300, 400 and 500 \mathrm{mmHg}$. At each $PO_{2}$ level, the corresponding values for $a_{1}, a_{2},a_{3} and a_{4}$ can be solved, yielding five $PO_{2}$-specific datasets in total.

Now consider the coefficient $a_{4}$​, as defined above:

|  | $a_{4}=\frac{\lambda_{i,4}\left( PO_{2} \right)^{i}}{\lambda_{i,0}\left( PO_{2} \right)^{i}}=\frac{\lambda_{0,4}}{1+\lambda_{1,0}\left( PO_{2} \right)^{1}+\lambda_{2,0}\left( PO_{2} \right)^{2}+\lambda_{3,0}\left( PO_{2} \right)^{3}+\lambda_{4,0}\left( PO_{2} \right)^{4}}$ | ----- Eq. 5 |
| --- | --- | --- |

With five pairs of $PO_{2}$​ values and their corresponding $a_{4}$​ values, substitution into equation (5) yields a system of five equations. This system contains five unknowns — $\lambda_{0,4}, \lambda_{1,0}, \lambda_{2,0}, \lambda_{3,0}, \lambda_{4,0}$ — and is therefore theoretically solvable. The solution provides the values of these association constants.

Next, consider the coefficient $a_{1}$.

|  | $a_{1}=\frac{\lambda_{i,1}\left( PO_{2} \right)^{i}}{\lambda_{i,0}\left( PO_{2} \right)^{i}}=\frac{\lambda_{0,1}+\lambda_{1,1}\left( PO_{2} \right)^{1}+\lambda_{2,1}\left( PO_{2} \right)^{2}+\lambda_{3,1}\left( PO_{2} \right)^{3}}{1+\lambda_{1,0}\left( PO_{2} \right)^{1}+\lambda_{2,0}\left( PO_{2} \right)^{2}+\lambda_{3,0}\left( PO_{2} \right)^{3}+\lambda_{4,0}\left( PO_{2} \right)^{4}}$ | ----- Eq. 6 |
| --- | --- | --- |

It is important to note that the denominator of equation (6) is identical to that of equation (5). In this case, only four unknowns — $\lambda_{0, 1}, \lambda_{1,1}, \lambda_{2,1}, \lambda_{3,1}$ — remain to be determined. Consequently, four pairs of $PO_{2}$ values and their corresponding $a_{1}$ values are sufficient to solve for these constants.

Next, consider the coefficient $a_{2}$:

|  | $a_{2}=\frac{\lambda_{i,2}\left( PO_{2} \right)^{i}}{\lambda_{i,0}\left( PO_{2} \right)^{i}}=\frac{\lambda_{0,2}+\lambda_{1,2}\left( PO_{2} \right)^{1}+\lambda_{2,2}\left( PO_{2} \right)^{2}}{1+\lambda_{1,0}\left( PO_{2} \right)^{1}+\lambda_{2,0}\left( PO_{2} \right)^{2}+\lambda_{3,0}\left( PO_{2} \right)^{3}+\lambda_{4,0}\left( PO_{2} \right)^{4}}$ | ----- Eq. 7 |
| --- | --- | --- |

As with equations (5) and (6), the denominator in equation (7) remains identical. In this case, only three unknowns — $\lambda_{0, 2}, \lambda_{1,2}, \lambda_{2,2}$ — need to be determined. Therefore, three pairs of $PO_{2}$ values and their corresponding $a_{2}$​ values are sufficient to solve for these constants.

Lastly, consider the following equation:

$$a_{3}=\frac{\lambda_{i,3}\left( PO_{2} \right)^{i}}{\lambda_{i,0}\left( PO_{2} \right)^{i}}=\frac{\lambda_{0,3}+\lambda_{1,3}\left( PO_{2} \right)^{1}}{1+\lambda_{1,0}\left( PO_{2} \right)^{1}+\lambda_{2,0}\left( PO_{2} \right)^{2}+\lambda_{3,0}\left( PO_{2} \right)^{3}+\lambda_{4,0}\left( PO_{2} \right)^{4}}$$

In this case, there are only two unknows: $\lambda_{0, 3}, \lambda_{1,3}$. Therefore, only two pairs of $PO_{2}$ values and their corresponding $a_{3}$ values are sufficient to solve for these constants.

Hence, the general equation of carbon monoxide hemoglobin association can be expressed as:

$$CO sat=\frac{a_{1}\left( PCO \right)+2a_{2}\left( PCO \right)^{2}+3a_{3}\left( PCO \right)^{3}+4a_{4}\left( PCO \right)^{4}}{4+4a_{1}\left( PCO \right)+4a_{2}\left( PCO \right)^{2}+4a_{3}\left( PCO \right)^{3}+4a_{4}\left( PCO \right)^{4}}$$

where

$$a_{j}=\frac{\lambda_{i,j}\left( PO_{2} \right)^{i}}{\lambda_{i,0}\left( PO_{2} \right)^{i}}, 0\leq i\leq4, 0\leq j\leq4, 0\leq i+j\leq4$$

This formulation incorporates both PO_2_ ​and PCO, allowing calculation of carboxyhemoglobin saturation under varying oxygen and carbon monoxide partial pressures.
